# Cocktail party training induces increased speech intelligibility and decreased cortical activity in bilateral inferior frontal gyri. A functional near-infrared study

**DOI:** 10.1371/journal.pone.0277801

**Published:** 2022-12-01

**Authors:** Cosima Lanzilotti, Guillaume Andéol, Christophe Micheyl, Sébastien Scannella

**Affiliations:** 1 Département Neuroscience et Sciences Cognitives, Institut de Recherche Biomédicale des Armées, Brétigny sur Orge, France; 2 ISAE-SUPAERO, Université de Toulouse, Toulouse, France; 3 Thales SIX GTS France, Gennevilliers, France; 4 Starkey France, Creteil, France; Universidad de Chile, CHILE

## Abstract

The human brain networks responsible for selectively listening to a voice amid other talkers remain to be clarified. The present study aimed to investigate relationships between cortical activity and performance in a speech-in-speech task, before (Experiment I) and after training-induced improvements (Experiment II). In Experiment I, 74 participants performed a speech-in-speech task while their cortical activity was measured using a functional near infrared spectroscopy (fNIRS) device. One target talker and one masker talker were simultaneously presented at three different target-to-masker ratios (TMRs): adverse, intermediate and favorable. Behavioral results show that performance may increase monotonically with TMR in some participants and failed to decrease, or even improved, in the adverse-TMR condition for others. On the neural level, an extensive brain network including the frontal (left prefrontal cortex, right dorsolateral prefrontal cortex and bilateral inferior frontal gyri) and temporal (bilateral auditory cortex) regions was more solicited by the intermediate condition than the two others. Additionally, bilateral frontal gyri and left auditory cortex activities were found to be positively correlated with behavioral performance in the adverse-TMR condition. In Experiment II, 27 participants, whose performance was the poorest in the adverse-TMR condition of Experiment I, were trained to improve performance in that condition. Results show significant performance improvements along with decreased activity in bilateral inferior frontal gyri, the right dorsolateral prefrontal cortex, the left inferior parietal cortex and the right auditory cortex in the adverse-TMR condition after training. Arguably, lower neural activity reflects higher efficiency in processing masker inhibition after speech-in-speech training. As speech-in-noise tasks also imply frontal and temporal regions, we suggest that regardless of the type of masking (speech or noise) the complexity of the task will prompt the implication of a similar brain network. Furthermore, the initial significant cognitive recruitment will be reduced following a training leading to an economy of cognitive resources.

## Introduction

Our ability to selectively attend to a specific sound source in the presence of other sounds has been a research topic of interest to psychologists and neuroscientists for several decades [1–6]. An everyday-life example of this ability can be found in multi-talker conversations where extracting information from a target speech is challenging. Several previous studies have started to elucidate psychoacoustical, cognitive, and neurobiological processes involved in the ability to selectively attend to and decode a speech stream in the presence of concurrent streams, including competing voices [7, 8]. Broadly, auditory stream segregation and selective auditory attention likely play a crucial role for good speech intelligibility in such listening situations [9]. Psychoacoustic studies have outlined various acoustic cues, such as fundamental-frequency, sound-intensity, or source-location differences between the target and competing voices [10–13], which can be used in auditory and cognitive systems to separate concurrent voices, and to group or ‘track’ over time sounds from a given voice.

In parallel to these behavioral investigations, brain-imaging studies have started to shed light on brain networks involved in speech comprehension in adverse listening situations. From the recent review of Peelle [9], it emerges that the quality of an auditory stimulus (acoustically clear or degraded) can determine the type of the cognitive recruitment required to perform the listening task. In particular, an acoustically clear auditory stimulus should involve the recruitment of a Core Speech Network, including the left inferior frontal gyrus (IFG) and bilateral temporal cortices. When speech is degraded, this core speech network shows increased neural activation and additional brain regions are recruited in order to deal with the increased difficulty in extracting the auditory information. The latter brain regions comprise the cingulo-opercular cortex, the premotor cortex, the dorsolateral prefrontal cortex (DLPFC) and the inferior parietal cortex [9, 14–17].

Recent studies have talked the question of underlined neural substrate of speech-in-speech paradigm within fMRI scanners [18, 19]. In Evans and colleagues’ study [18], the authors used target narratives with or without the presence of competing speech and assessed intelligibility performance after the participants completed the task. Their results partially support speech-in-noise findings as they found increased activation for masked *versus* clear speech beyond the temporal lobe, in regions associated with cognitive control (i.e., anterior cingulate, middle frontal gyri and insula, left inferior and superior parietal lobule and right inferior frontal gyrus). According to the authors themselves however, the sound of the imaging acquisition within the scanner is a significant issue for sound processing that is likely to interfere with auditory and cognitive processes, making the results poorly transferable to real life situations. In addition, the fact that the behavioral response was assessed after the task–outside the scanner–arguably involved additional working memory effort to some extent.

To study spoken language in a more ecological way, some researchers have started to use optical imaging that has the advantage of being silent, albeit covering only the surface of the cortex when used in conventional designs [9, 20]. For instance, using a speech-in-speech paradigm, Andéol et al. [21] showed a positive correlation between prefrontal activity and speech intelligibility in the most difficult condition, reflecting greater recruitment of cognitive resources outside the core speech network. Consistently with these findings, it has been suggested that in a complex auditory scene at least two high-level cognitive processes, namely the working memory and attentional-based functions [22–24], are involved in selecting targeted auditory stream thanks to the information provided by the low level processes [9]. Regarding the attentional-based functions specifically, it seems that individual inhibitory control capacity–as assessed by the Stroop task [25] for instance–plays a significant role in the target voice selection [26–28]. This hypothesis is furthermore supported by the fact that errors in speech-in-speech paradigms often result from participants reporting the masker’s words instead of the target’s ones [29] contrary to speech-in-noise paradigms.

Difficulties however, in interpreting the results from the literature, stem at least from two principal factors. First, according to Evans et al. [18], research in speech perception should take into consideration the distinction between energetic masking (i.e., interferences between sounds at the energetic level in terms of power) and informational masking (i.e., interferences between sounds that share perceptual attributes that can draw attention). Indeed, as the energetic masking reflects direct interaction within the auditory periphery, informational masking refers to additional effects underpinned by higher cognitive processes such as selection, inhibition and linguistic processing [19, 29–31]. As a consequence, energetic masking is likely to affect speech perception linearly, which is not the case for informational masking [29]. In addition, speech intelligibility in informational masking may improve over time, whereas it is relatively stable for energetic masking due to steady state noise [32]. Second, in multi-talker situations, speech intelligibility can be influenced by several factors. Some of them are related to low level processes–such as physical stimulus (acoustic) or the differences between the target and the masker voices–and others are more related to high level processes–such as attention and working memory [2, 9, 33, 34]. Therefore, the cortical activity recorded during a speech intelligibility task could be related to any or all of these low and high levels factors. However, as speech-in-speech performance is supposed to be more related to high level processes [34–37], there is a need in facilitating the recording of the associated brain networks. This could be achieved for instance, by keeping constant the acoustic characteristics of the stimuli handled by low levels processes, while training the listener in a goal to improve the efficiency of high-level processes.

The fact that speech intelligibility can improve with training is already well established [38–41]. Moreover, it is known that changes in strategies and improvements in performance in multi-talker speech-intelligibility tasks can also be achieved by providing participants with specific instructions and/or feedback [36, 42].

The present study takes advantage of such an approach based on inter-individual differences in, and intra-individual changes of, cognitive ability to address brain networks involved in performance of a speech-intelligibility task with two concurrent speech streams. Specifically, this approach builds upon earlier observations that some listeners use the sound-level difference between the target and the masker to aid in the identification and the segregation of them [21, 29, 36, 37, 43]. However, many listeners seem to not use this strategy, as shown by their poor performance in adverse target-to-masker ratio (TMR) [21]. We posited that listeners’ ability to exploit this strategy could be enhanced via the provision of specific instructions, practice (perceptual training) and feedback to participants in a training paradigm. This way, we were able to study changes in brain activity related specifically to changes in task performance, unconfounded by concomitant physical-stimulus changes.

Using the speech-in-speech Coordinate Response Measure (CRM) task [44], we postulated that brain areas outside the temporal lobe will be involved to ensure speech intelligibility in multi-talker situation in addition to the Core Speech Network like for the degraded speech [9]. More precisely, we targeted this additional network by probing the prefrontal cortex, the bilateral IFGs and the DLPFCs, the inferior parietal cortices and the auditory cortices. To prevent from any additional auditory interference, we used a functional near infrared spectroscopy (fNIRS) device in a quiet room. Also, occipital regions, for which conditions effects were not expected, have been probed in order to maximize specificity.

The study consisted of two experiments. In Experiment I (observational phase), initially naïve participants performed a speech-in-speech intelligibility task while their cortical activity was recorded. This first experiment had two purposes. The first one was to select participants that were not able to properly use the TMR as an auditory clue to segregate the auditory stream. These participants would then be involved in a training protocol designed to improve their use of this auditory clue. The second purpose was to reproduce previous results [21], hence we predicted that participants’ speech intelligibility scores in an adverse TMR condition would positively co-vary with frontal regions activity [21]. In Experiment II (training phase), participants who had the poorest speech-intelligibility scores in Experiment I were recruited and trained (through instructions, task practice, and feedback) to take advantage of sound-level differences between the target and masker voices. We predicted that, following this training, speech intelligibility would improve and that IFG, IPL and DLPFC cortical activities would decrease concomitantly reflecting a more efficient recruitment of cognitive resources implicated in inhibitory control [41, 45].

## Materials and methods

### Participants

Seventy-four adult volunteers (20 women, mean age = 23.6 ± 3.2 years) were recruited to take part in the study. All were students from ISAE-SUPAERO and Toulouse University with normal hearing, defined as pure-tone hearing thresholds better than 20 dB hearing level (HL) at octave frequencies from 125 Hz to 8 kHz (Audio Console Oscilla^®^ headphones with built-in software). The study was cleared by the local ethics committee (Comité de Protection des Personnes Sud-Ouest et Outre Mer II, IDRCB 2017-A00859–44). Before starting the study, all participants completed a written consent form. They were paid 15 euros for their participation in Experiment I. Participants who additionally completed Experiment II received a total of 60 euros.

### Speech stimuli

Stimuli used in the speech-intelligibility task were sentences from the CRM corpus [44]. CRM sentences are all constructed using the same pattern: “Ready *call sign* go to *color number* now*”*, where *call sign*, *colo*r, and *number* are placeholders. The actual call sign, color, and number played during a sentence are drawn from a list of 8 call signs (*Baron*, *Charlie*, *Ringo*, *Eagle*, *Arrow*, *Hopper*, *Tiger*, *Laker*), 4 colors (*red*, *blue*, *green*, *white*) and 8 numbers (from ‘one’ to ‘eight’) resulting in 256 possible sentences. The sentences used in this study were uttered by four male speakers.

For the purpose of the present study, the sentences were always presented in pairs, with one target sentence indicated by the call sign, *Baron*, and a concurrent (simultaneously presented) masker sentence drawn at random, and independently, from the list of the 7 remaining call signs. Three conditions, corresponding to three different TMRs, were tested: an ‘adverse’ condition (TMR = -12 dB), an ‘intermediate’ condition (TMR = -4 dB), and a ‘favorable’ condition (TMR = +4 dB). Stimuli corresponding to one of the three conditions were presented consecutively to the participant in a block of 32 pairs of sentences, before the next condition was tested, also as a block. The testing order of the three conditions was randomized across participants. Each stimulus (a pair of simultaneous sentences) lasted 2 s. After each stimulus, participants had to report the color and the number corresponding to the target sentence. They provided answers using a home-made response box. To allow sufficient time for measurement of the hemodynamic response, a minimum inter-stimulus-interval (ISI) of 18 s was enforced.

Stimuli (.wav audio files) were selected from the CRM corpus using custom software written in Matlab^®^ (2018b) on a Dell Optiflex 990 computer. All sounds were normalized at the same RMS level. Then, for each trial, a gain or attenuation was applied to the target sound depending on the selected TMR (-12, -4 or +4 dB). Both target and masker sounds were transmitted to a digital signal processor (RX8; Tucker Davis Technologies, TDT) to be filtered in real time with HRTFs (Head Related Transfer Functions) measured using a dummy head (Neumann KU-100). The target and masker sentences were filtered by the same frontal HRTF, such that they appeared to the listener to both be coming from straight ahead (0 degree azimuth and elevation). Then, target and masker sounds were added together, and, following the procedure of [29], a gain was randomly chosen over a 6-dB range (in 1-dB steps) to be applied on the sound mixture. The signal was pre-amplified (HB7, TDT) and presented binaurally through headphones (Beyerdynamic DT770) at an overall level of approximately 70 dB SPL. Behavioral responses were recorded using a homemade response box with three rows of buttons: a first row for the colors, a second row for numbers one to four, and a third row for numbers five to eight. The response box was linked to the real-time processor and the responses were recorded with Matlab^®^.

### Experimental design

This study included two experiments. A first experiment (Experiment I) involved all 74 participants. This was followed by a second experiment (Experiment II), which involved only a subset of participants from Experiment I, selected based on their performance in the adverse condition of Experiment I. Experiment II involved a training program over three consecutive days (T1, T2, T3), followed by one final test session, which was similar to the single test session of Experiment I.

### fNIRS data collection

**Fig 1:**
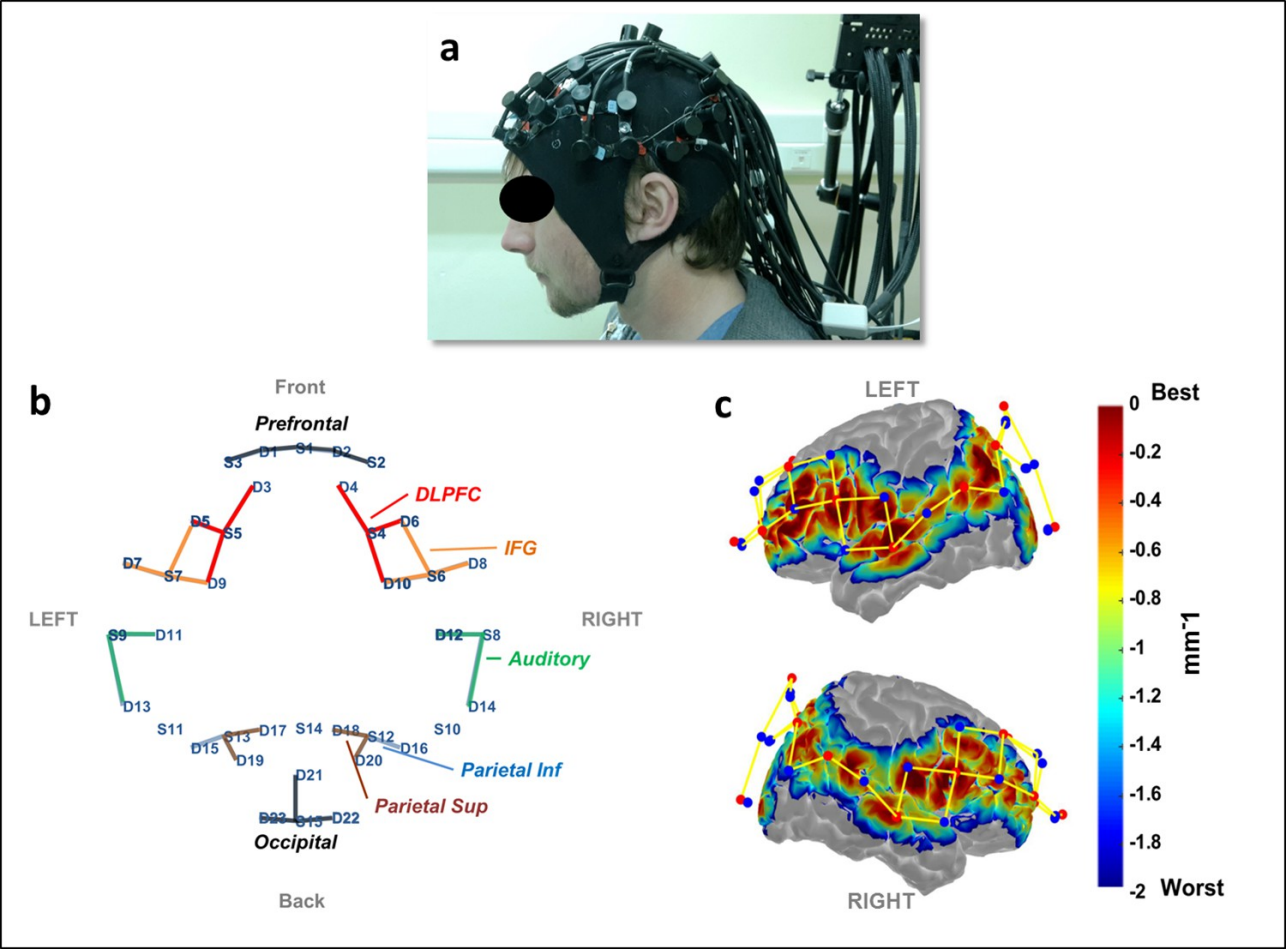
Graphic representation of fNIRS data collection. a) Photography of the optode-array holder placed on the head of one of the participants for demonstration purposes. (b) 2D representation of ROI location; each line represents a measurement channel between source (S) and detector (D) optodes. (c) Left (up) and right (down) views of the cortical sensitivity maps obtained using Monte-Carlo simulation within Atlas Viewer (V2.8; default parameters except for the number of photons that has been set to 10^7^).

Cortical activity was assessed with a whole-head fNIRS device NIRScout (Nirx https://nirx.net/nirscout/). A 15-by-23 optode array, including 15 emitters (λ_1_ = 760 nm; λ_2_ = 850nm) and 23 detectors, yielding 44 measurement channels in total (see Fig 1B), was used. The source-detector spacing was maintained at approximately 30 mm using plastic spacers. The sampling rate was 7.81 Hz. Data were acquired using NIRSTAR v14.2 software. Channel positioning was made using the international 10–20 EEG system. The 15 sources were positioned at Fpz, AF7, AF8, F3, F4, FC5, FC6, T7, T8, TPP7h, TPP8h, P3, Pz, P4, Oz; the 23 detectors were positioned at Fp1, Fp2, AF3, AF4, F5, F6, FT7, FC3, FC4, FT8, C5, C6, TP7, TP8, P5, P1, P2, P6, PO1, POZ, PO2, O1, O2.

Cortical sensitivity maps were computed using a Monte-Carlo simulation algorithm within Atlas Viewer (V2.8) (Fig 1C). Default parameters were used, except for the number of photons, which was set to 10^7^ [46]. From the channel MNI coordinates obtained in Atlas viewer, regions targeted by our montage were localized with Talairach software [47]. In the Talairach Client, we used a 4 mm option to get a description of the brain regions within a 9 mm^3^ volume around each channel coordinate as the spatial resolution of fNRIS provides a brain observation area of roughly 1 cm^2^ around the center of the Montreal Neurological Institute (MNI) coordinate system [48]. Finally, the sub-regions have been concatenated across the channels that belong to the same functional region.

As a result, thirteen brain region of interests (ROIs) were defined (Fig 1B and Table 1), corresponding to: Prefrontal Cortex (PFC), Dorsolateral Prefrontal Cortex (DLPFC), Inferior Frontal Gyrus (IFG), Auditory Cortex (AC), Superior Parietal Lobe (SPL), Inferior Parietal Lobe (IPL), and Visual Cortex (V1). The regions listed are to be considered bilaterally except for V1 that was considered as a unique ROI.

**Table 1:** Description of fNIRS ROIs and measurement channels.

ROI	Left Hemisphere					Right Hemisphere				
	Channels		MNI Coordinates			Channels		MNI Coordinates		
	Source	Detector	x	y	z	Source	Detector	x	y	z
**PFC**	S1	D1	-12	72	1	S1	D2	15	70	0
	S3	D1	-25	58	2	S2	D2	36	67	5
**DLPFC**	S5	D3	-23	49	24	S4	D4	23	44	26
	S5	D9	-40	32	31	S4	D10	46	23	32
	S5	D5	-36	35	18	S4	D6	50	-40	-25
**IFG**	S7	D7	-42	9	16	S6	D8	48	9	10
	S7	D9	-57	24	34	S6	D10	46	13	25
	S7	D5	-55	34	22	S6	D6	46	24	19
**AC**	S9	D11	-50	-12	9	S8	D12	64	-10	12
	S9	D13	-58	-20	8	S8	D14	51	-20	12
**SPL**	S13	D17	-33	-69	54	S12	D18	39	-70	53
	S13	D19	-36	-76	47	S12	D20	39	-75	44
**IPL**	S13	D15	-50	-12	9	S12	D16	43	-64	33
**V1**	S15	D23	-10	-97	6	S15	D22	20	-99	7
						S15	D21*	3	-96	24

MNI coordinates (x, y, z of the centroids) extracted from Atlas viewer (V2.8).

*The S15-D21 channel is actually centered between the two hemispheres but is presented here as part of the right hemisphere for simplicity purposes. PFC: Prefrontal Cortex; DLPFC: Dorsolateral Prefrontal Cortex; IFG: Inferior Frontal Gyrus; AC: Auditory Cortex; SPL: Superior Parietal Lobule; IPL: Inferior Parietal Lobule; V1: Primary visual cortex.

### fNIRS preprocessing

**Fig 2:**
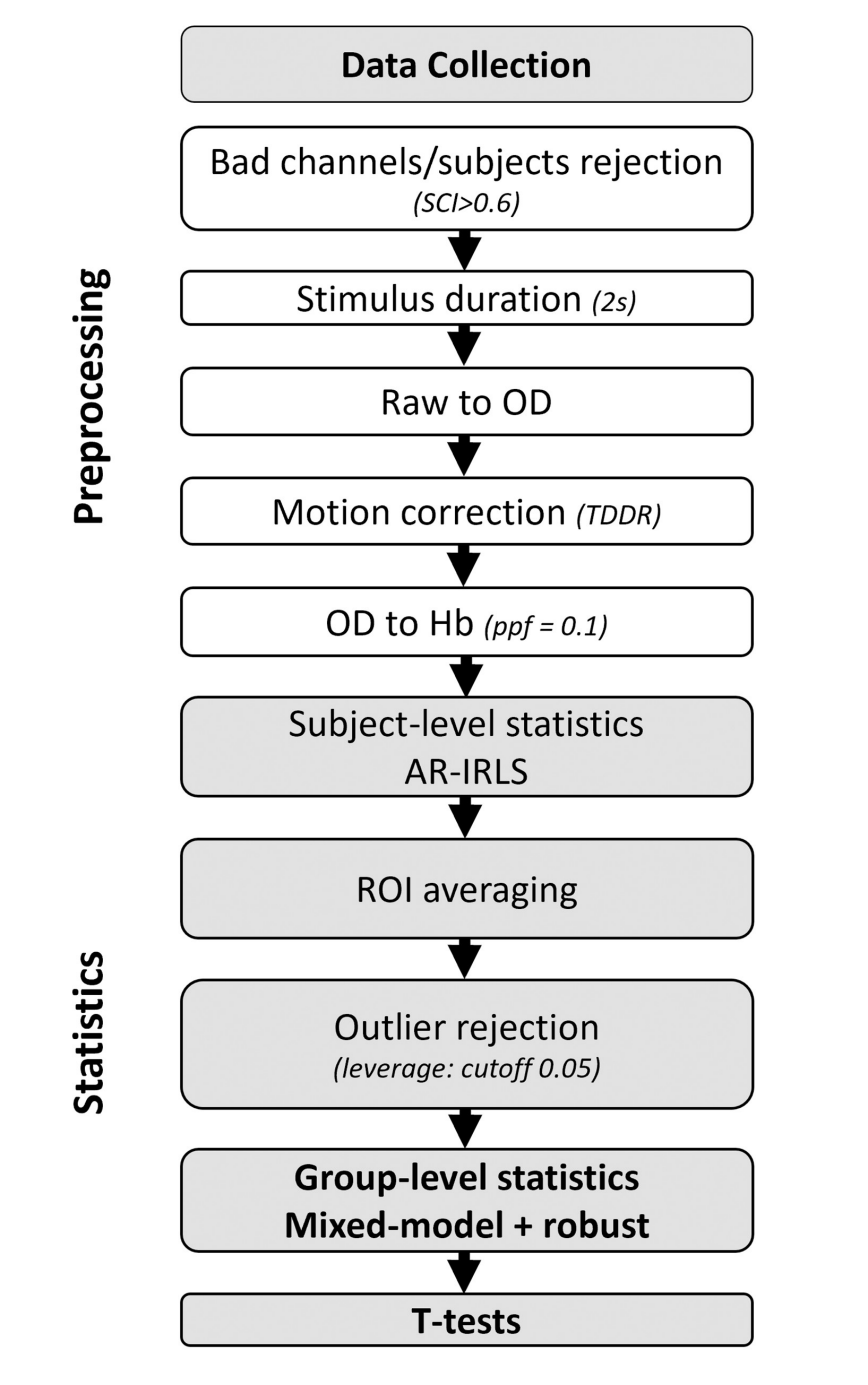
fNIRS data collection, processing and statistical analysis pipeline. Accurate description of all phases of data acquisition, data processing (rejection bad channels, filtering, data conversion) and subject and group level statistical analyses.

Preprocessing and statistical analyses are summarized in Fig 2. Data were processed using the Matlab-based NIRS AnalyzIR toolbox [49] V.615, with Matlab^®^ R2018b. First, raw fNIRS data were imported in Matlab^®^ and a demographic table was created based on the NIRstar demographic inputs and on task-performance results. Channel quality check was carried out using the Scalp Coupling Index (SCI) procedure [50]. Channels with SCI-value of +0.60 or greater were kept for further analyses. We considered a ROI reliable enough for analyses when at least n-1 channels within this ROI were above the +0.60 threshold (i.e., 2 channels > 0.60 for a 3-channel ROI, 1 channel > 0.60 for a 2-channel ROI and 1 channel > 0.60 for a 1-channel ROI). If these criteria were not reached, the whole participant’s data were excluded for further analyses. For experiment I, over the 74 participants, 11 were thus excluded. For the 63 remaining ones, 2.5 channels (±3) were excluded. Stimulus duration was set to 2s, corresponding to the duration of CRM sentences. Raw data were then converted into optical density and submitted to a Temporal Derivative Distribution Repair (TDDR) for motion correction [51]. These authors have shown that this correction is highly effective in correcting movement artifacts in fNIRS data. Concentration changes for oxygenated and deoxygenated hemoglobins were computed using the modified Beer-Lambert law with a partial path length factor (ppf) of 0.1 for both wavelengths.

## Experiment I: Naive participants

### Methods

#### Participants

Participants in Experiment I performed the concurrent-speech intelligibility task described above while their brain activity was recorded with the 15-by-23 optodes NIRScout fNIRS device (Nirx https://nirx.net/nirscout/). Before fNIRS data acquisition, all participants performed one short training block of 16 speech stimuli at 0 dB TMR to verify correct understanding of the instructions and provide basic familiarization with the task. The whole Experiment I lasted approximately one hour.

#### Statistical analyses

*Behavioral analyses*. A response was considered as correct when both the color and the number were correctly reported. The correct response rate for each condition was transformed using the Rationalized Arcsin Unit (RAU) transform [52]; this transform yields results more suitable for parametric statistical analyses than untransformed percent-correct data.

A preliminary Mauchly’s Sphericity test came up significant for the TMR factor (χ2 = 11.24; *p* < 0.01), hence a multivariate analysis of variance (MANOVA) was used to test for condition effect over the RAU scores, with TMRs (Adverse *vs*. Intermediate *vs*. Favorable) as a within-subject factor. Post-hoc comparisons were carried out using the Tukey’s Honest Significant Difference (HSD) test. All behavioral statistical analyses were performed using the software STATISTICA^TM^ (V10) from StatSoft® and a significant threshold was set to 0.05.

*fNIRS—task activation*. Task activation was quantified by convolving the preprocessed HbO (Oxygenated hemoglobin) signal with the hemodynamic response function (HRF) referred to as “canonical” within the Nirstoolbox for each condition (Adverse, Intermediate and Favorable TMRs). The canonical HRF was used with the default parameters (*uShootTime = 16s; ratio = ⅙; duration = 32s*), except for the *peakTime* that has been changed from 4 to 5s according to Pedregosa et al. [53]. Following Barker et al. [54], beta coefficients were then estimated using an autoregressive iteratively reweighted least squares (AR-IRLS) algorithm; this algorithm was used to account for the presence of serial correlations in fNIRS data and to control for Type-1 errors. As a consequence, and following Huppert’s recommendations [55], no additional filtering or prewhitening of the data has been applied to avoid bias in the estimated response. The estimated betas for the three conditions were then submitted to a robust mixed-effects model (see Eq 1, according to Wilkinson notation [56] in the NirsToolbox code), with condition as a fixed effect (Cond) and subject as a random effect (1|Subject) using the *nirs*.*modules*.*MixedEffects* function of the toolbox.


beta∼−1+Cond+(1|Subject) (1)

The *robustfit* function with the bisquare weight function and default tuning constant value (i.e., 4.685) was used by selecting the option *robust = true* within the *nirs*.*modules*.*MixedEffects* function.

Lastly, statistical differences in the weighted betas across the 3 TMR conditions (Adverse *vs*. Intermediate *vs*. Favorable) were assessed using multiple two-sample t-tests. Multiple comparisons across ROIs were controlled for using the Benjamini-Hochberg procedure [57], and *p*-values were corrected using the false discovery rate (FDR), corresponding to the *q-values* in the toolbox output.

*Relationship between cortical activity and speech intelligibility scores*. The relationship between speech intelligibility scores and brain activations across ROIs was assessed using second-level robust mixed-effects models for each condition separately, with condition as a fixed effect (Cond) and speech-intelligibility scores (RAU) as regressors (see Eq 2). As for the task activation, it uses the *robustfit* function from Matlab^®^ with the bisquare weight function and default tuning constant value (i.e., 4.685). As for the task activation, multiple comparisons across ROIs were controlled for using the Benjamini-Hochberg procedure [57], and *p*-values were corrected using the false discovery rate (FDR).


beta∼−1+Cond+Cond:RAU (2)

### Results

#### Behavior

**Fig 3:**
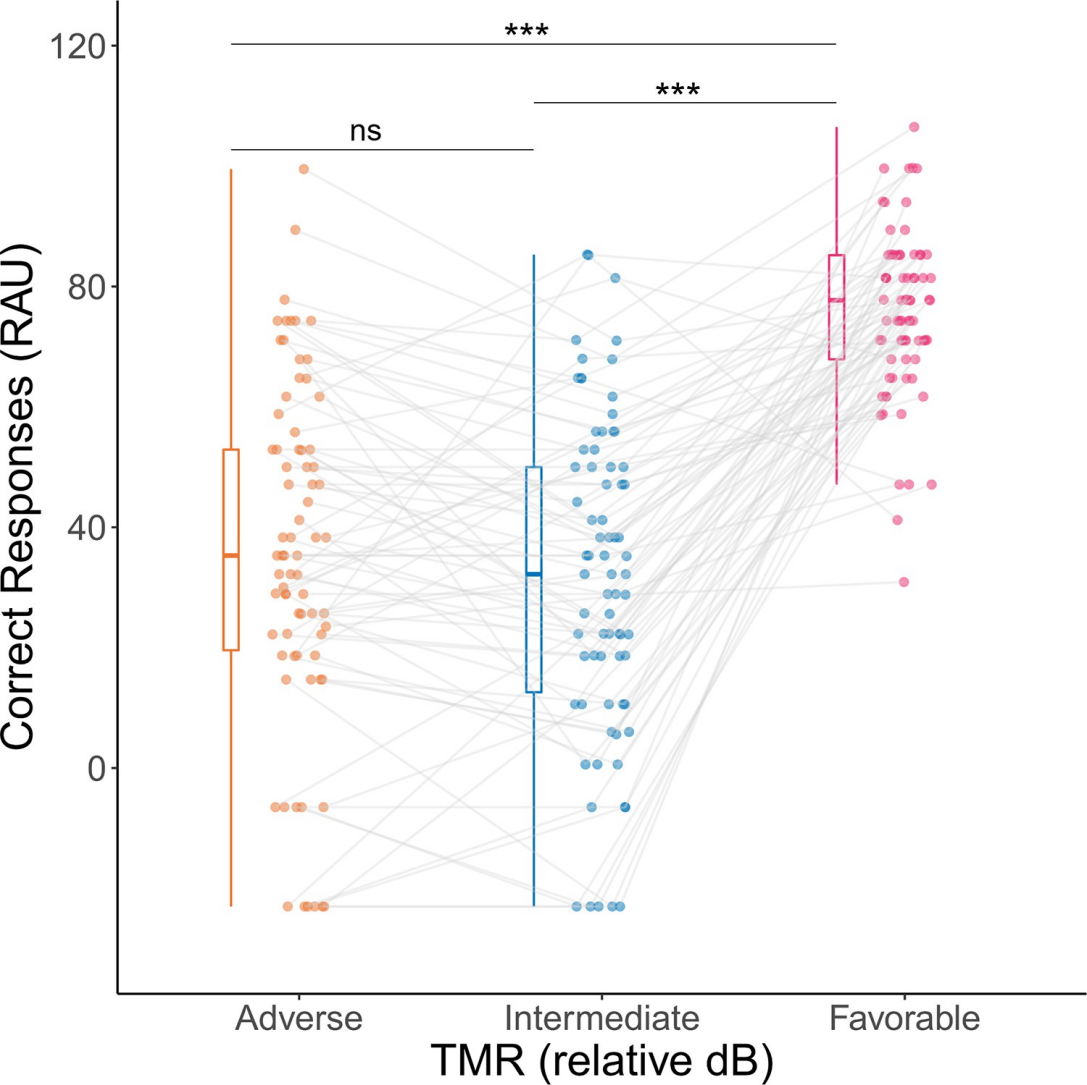
Correct responses (Rationalized Arcsin Units) as a function of TMR in Experiment I. Boxplots show the median (horizontal bar) and the interquartile range (boxes). The whiskers reach from the lowest to the highest observed value within 1.5 times the interquartile range. Dots show individual data points. Individual data points corresponding to the same listener across different conditions are connected with grey lines. ***: *p* < 0.001.

Behavioral data showed that the TMR level had a significant effect on the target speech intelligibility (F(2, 146) = 90.63, *p* < 0.001; see Fig 3). This corresponded to higher scores with the favorable TMR (M = 76.03; SD = 14.93), compared to the intermediate (M = 31.56; SD = 26.85; *p* < 0.001) and the adverse ones (M = 35.37; SD = 28.95; *p* < 0.001). No statistically significant difference however was observed between the intermediate and adverse TMRs (*p* = 0.5). These data also revealed a large variability in the ability to use the TMR as a clue to segregate the two voice streams with the largest standard deviation in the adverse condition (SD = 35.37) followed by the intermediate one (SD = 31.56).

#### fNIRS

**Fig 4:**
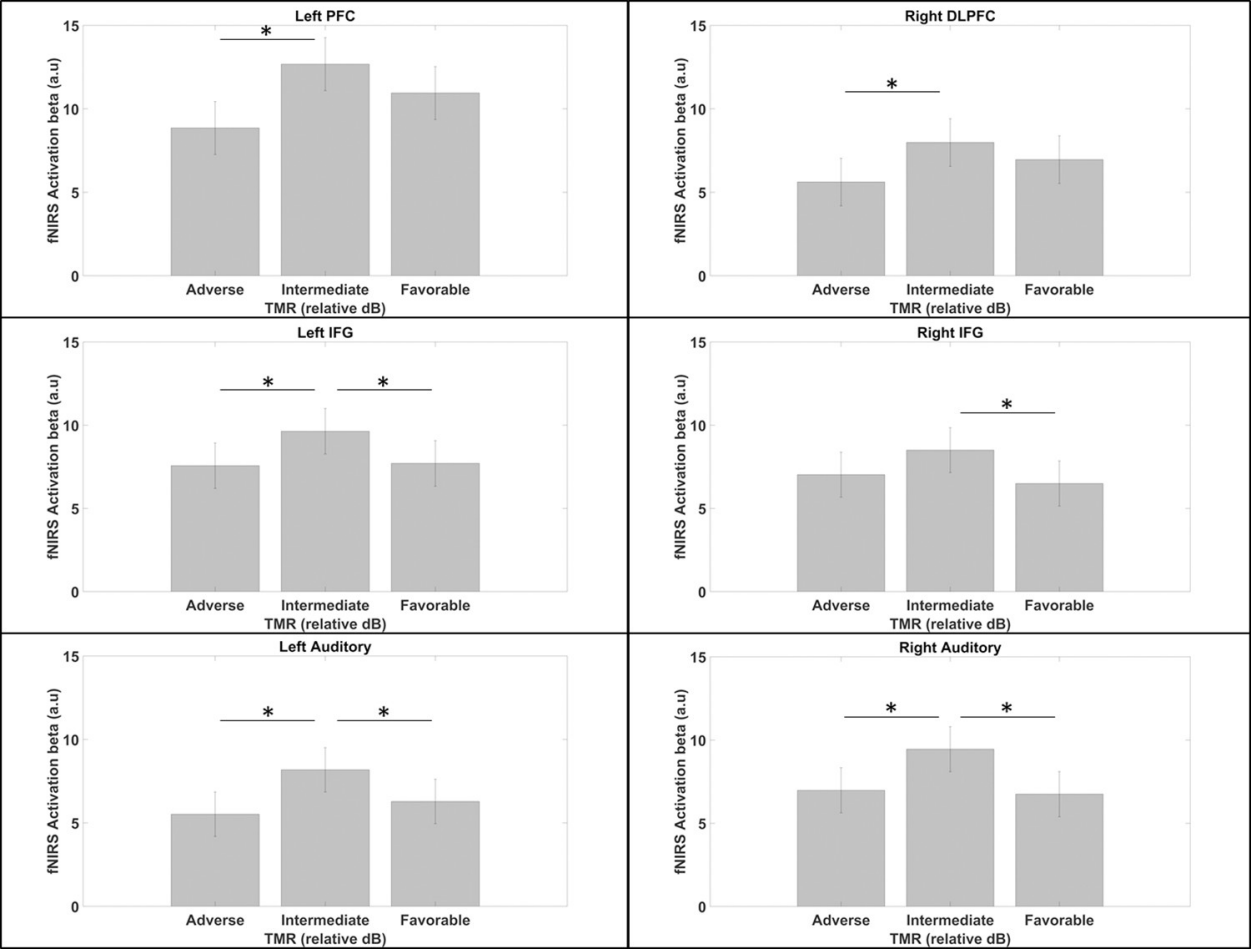
HbO activations for the TMR condition effect (t-test results). *: *p* < 0.05 (FDR-corrected) for the number of ROI areas. Vertical bars denote the standard error of the mean.

*Task activation*. Fig 4 illustrates the main significant findings. We found that greater activation was observed for the intermediate-TMR condition compared to the adverse-TMR condition in frontal ROIs (left PFC, left IFG and right DLPFC; *p* < 0.05; FDR-corrected) and temporal regions (bilateral auditory cortex: *p* < 0.05; FDR-corrected). Greater activation was also observed for the intermediate-TMR condition compared to the favorable-TMR condition in bilateral IFGs and auditory cortices (*p* < 0.05; FDR-corrected). No other contrast in any region showed significant result.

**Fig 5:**
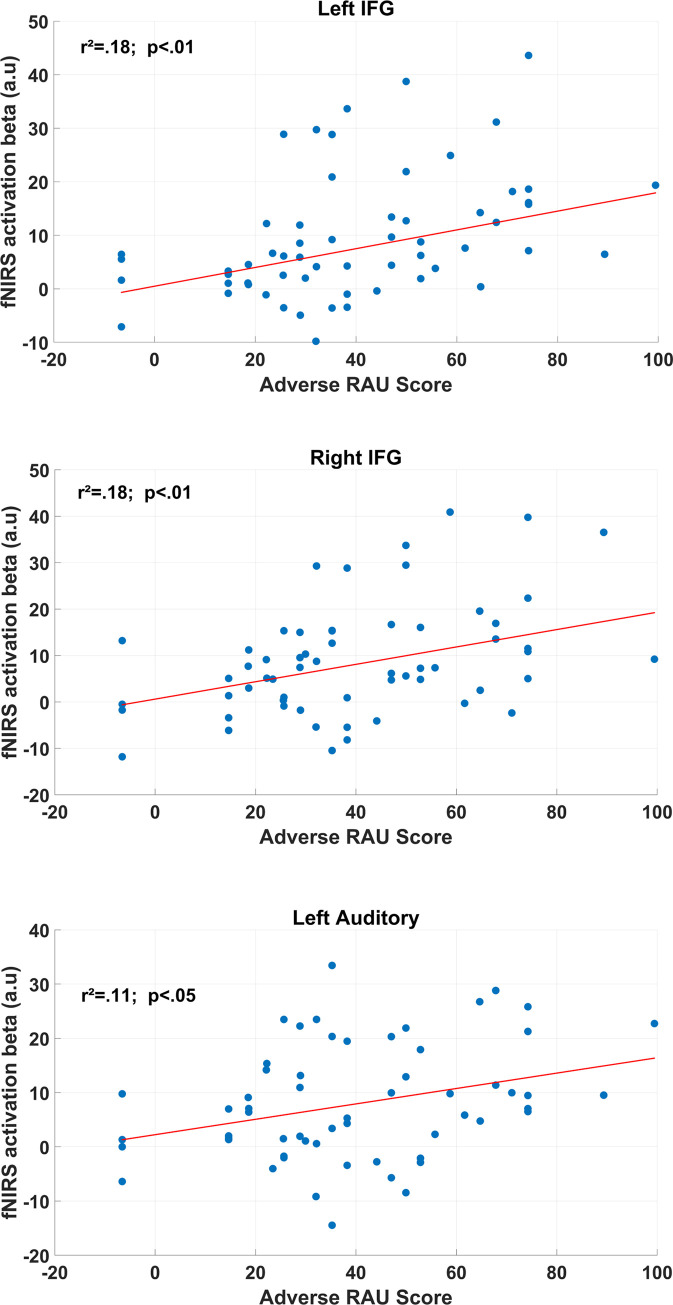
Relationship between cortical activity (beta coefficients for HbO) and speech intelligibility (RAU scores) for the adverse condition. Scatter plot of the robust linear regression results for the left and right IFGs and left auditory cortex. The red line indicates the robust linear fit. Reported *p*-values are FDR corrected.

*Relationship between cortical activation and intelligibility scores*. To confirm the relationship between the cortical activation and the behavioral performance, a robust regression was carried-out using the weighted betas (i.e., cortical activation) as the dependent variable and the RAU score (i.e., behavioral accuracy) as the independent variable (see Fig 5 for main results). For the adverse-TMR condition, statistically significant positive correlations between these two variables were observed for the left auditory cortex (r^2^ = 0.11; *p* < 0.05), and the bilateral IFGs (r^2^ = 0.18; *p* < 0.01, for both correlations). In these regions, the higher the speech intelligibility, the higher the cortical activity. Similarly, a positive correlation was also found within the right IFG in the favorable-TMR condition (r^2^ = 0.09; *p* < 0.05). For the intermediate condition the same robust-regression analyses failed to show statistically significant linear associations between RAU scores and cortical activation.

### Discussion I

In Experiment I, a speech-in-speech listening task was used to explore the relationship between speech intelligibility and cortical activity. As previously shown, intelligibility at the group level was higher in the favorable TMR but did not decrease from intermediate to adverse TMR. However, large individual differences revealed that some listeners were able to reach more than half of correct responses in the adverse condition whereas others did not (Fig 3). More precisely, for some listeners, speech intelligibility improved as the difference in sound level between the target and masker voices (i.e., the target-to-masker ratio, TMR) increased from adverse (negative) to favorable (positive). For other listeners, intelligibility was actually highest for adverse and favorable TMRs, whereas it was very low for intermediate TMRs–that is, when the target and masker voices had approximately the same sound level [21, 29, 36, 37, 43]. Although the latter finding may initially seem counter-intuitive, it can easily be accounted for by considering that, when the target and masker voices have different sound levels, listeners can in principle take advantage of these differences to aid in the identification of target and masker sounds. However, this strategy is only available to listeners who perceive the sound-level difference and who are actually able to use it as an advantage. The latter result fulfilled our first purpose which was to select participants who were not able to properly use this auditory cue and would be then involved in a training protocol designed to improve their use of it.

#### Task activation

While there was no difference between the adverse and intermediate conditions at the behavioral level, there was a clear difference at the neural level between conditions. Indeed, greater activation was observed within the left PFC, the bilateral IFG, the right DLPFC and the bilateral STG for the intermediate-TMR condition compared to the adverse-TMR condition and the favorable-TMR condition. As discussed above, the intermediate-TMR condition is an *a priori* intermediate difficulty condition given that several participants reached higher speech intelligibility scores in the adverse condition compared to the intermediate one. The significant higher cortical activations for the intermediate-TMR condition are thus in favor of a global higher mental demand for this condition.

The scientific literature of listening effort has already shown a significant non-linear relationship between cortical activity and the intrinsic complexity of the task in terms of intelligibility of the stimulus to be processed. Notably, the involvement of the left IFG in complex listening situations was greater when the signal was degraded but still intelligible compared to situations in which the signal was perfectly intelligible, or in non-intelligible noise conditions [15–17, 58]. This activation would follow a so-called U-reverse activation with respect to the difficulty of the task. This non-monotonic relationship has also been observed in other objective markers of listening effort, notably pupillary dilation [59]. Intermediate intelligibility conditions, compared to perfect or impossible intelligibility conditions, require effort to accomplish the task. In the intermediate intelligibility condition, the listener still remains motivated and feels able to succeed in the task [23, 60, 61]. On the other hand, if the task becomes too difficult (near impossible) or if the cost of completing the task is deemed too high [60], activation decreases, probably due to a disengagement. The particularity in our case is that this relative disengagement may have occurred only for some of the listeners, that is, those who were not able to use the unfavorable TMR to their advantage. To explore this result, we carried out regression analyses in this first experiment (see Fig 5).

#### Relationship between speech intelligibility and brain activations

The relationship between speech intelligibility scores and brain activation within each condition led to two main findings. Results suggest the involvement of i) a fronto-temporal network (bilateral IFG and left auditory cortex) in the adverse TMR condition and ii) the right IFG in the favorable TMR condition. Those results show that the situation is more complex than what task activation revealed. This positive correlation between cortical activity and performance is in favor of engagement variability in the task with some individuals remaining engaged in the task by trying to solicit more resources while other may have partially (e.g., for some trials) stop trying. As a result, the cortical activity variability in our sample was related to the capacity to use the TMR in the adverse condition, whereas other speech segregation mechanisms may have occurred in the intermediate one, like those based on voice frequency characteristics [2] for instance.

The literature has already established the role of the left IFG in speech-in-noise paradigms [14]. We suggest now that the left IFG is involved in speech intelligibility whatever the masking type: energetic (speech-in-noise) or informational (speech-in-speech) when the task is challenging.

Regarding the right IFG, speech intelligibility in informational masking arguably requires the inhibition of the masker talker to focus on the target talker. Previous study also found that the right IFG plays a central role in inhibition processing in a Stop-Signal task or Go/No-Go test for instance [62]. Indeed, inhibitory control is necessary to suppress irrelevant cognitive processes or motor plans [63] and maintaining and adapting goal directed behaviors in changing situations [64]. This ability relies on a right-lateralized fronto-basal brain network that includes the right IFG [62, 64, 65]. This would also apply to the favorable-TMR condition that remains a challenging task.

The literature about challenging listening conditions also highlights the implication of a larger fronto-temporo-parietal network, not limited to the bilateral IFG, for optimal speech intelligibility [66–69]. While the higher activation of the auditory cortex is more linked to the acoustic features of the stimulus, the prefrontal and parietal regions show a stimulus-independent activation [9, 70]. This suggests that the involvement of these brain areas can be attributed to higher order processes like attention, monitoring and memory [9, 21].

#### Providing further evidence of the relationship between behavior and cortical activity

Although this first experiment provides evidence of a link between speech intelligibility and cortical activation when the TMR can be used, this link remains only observational. A more effective way to test this relationship is to quantify the effect of the modification of one variable over the other. Taking this into account, we designed a training paradigm in Experiment II using an approach based on training and/or coaching. We hypothesized that task performance in the adverse-TMR condition would improve in participants who initially had not performed well in this condition. To do so we (a) specifically draw their attention to the voice level differences with instructions (e.g., “the target voice has a lower sound level than the masker voice”), or (b) simply let them to further practice the task. Importantly, any improvement in behavioral task performance and/or cognitive engagement, which might result from such interventions, could not be ascribed to a change in the physical characteristics of the signals since TMRs remained the same before and after interventions. Therefore, any change in cortical activity between before and after this intervention would likely be indicative of a true association between cortical activity and behavioral performance.

## Experiment II–trained participants

### Methods

#### Participants and training groups

Twenty-seven participants from Experiment I who achieved less than 50% of correct responses in the adverse condition were recruited to take part in Experiment II. These selection criteria have been chosen to address the following requirements. First, we had to select a condition in which the speech intelligibility variability and the room for improvement were the largest, which resulted in the exclusion of the favorable TMR condition. As our hypotheses focused on the improvement in the use of TMR to better segregate the target voice, we selected the condition that most segregates between participants who are able to use it and those who are not (i.e., a TMR of -12dB).

**Fig 6:**
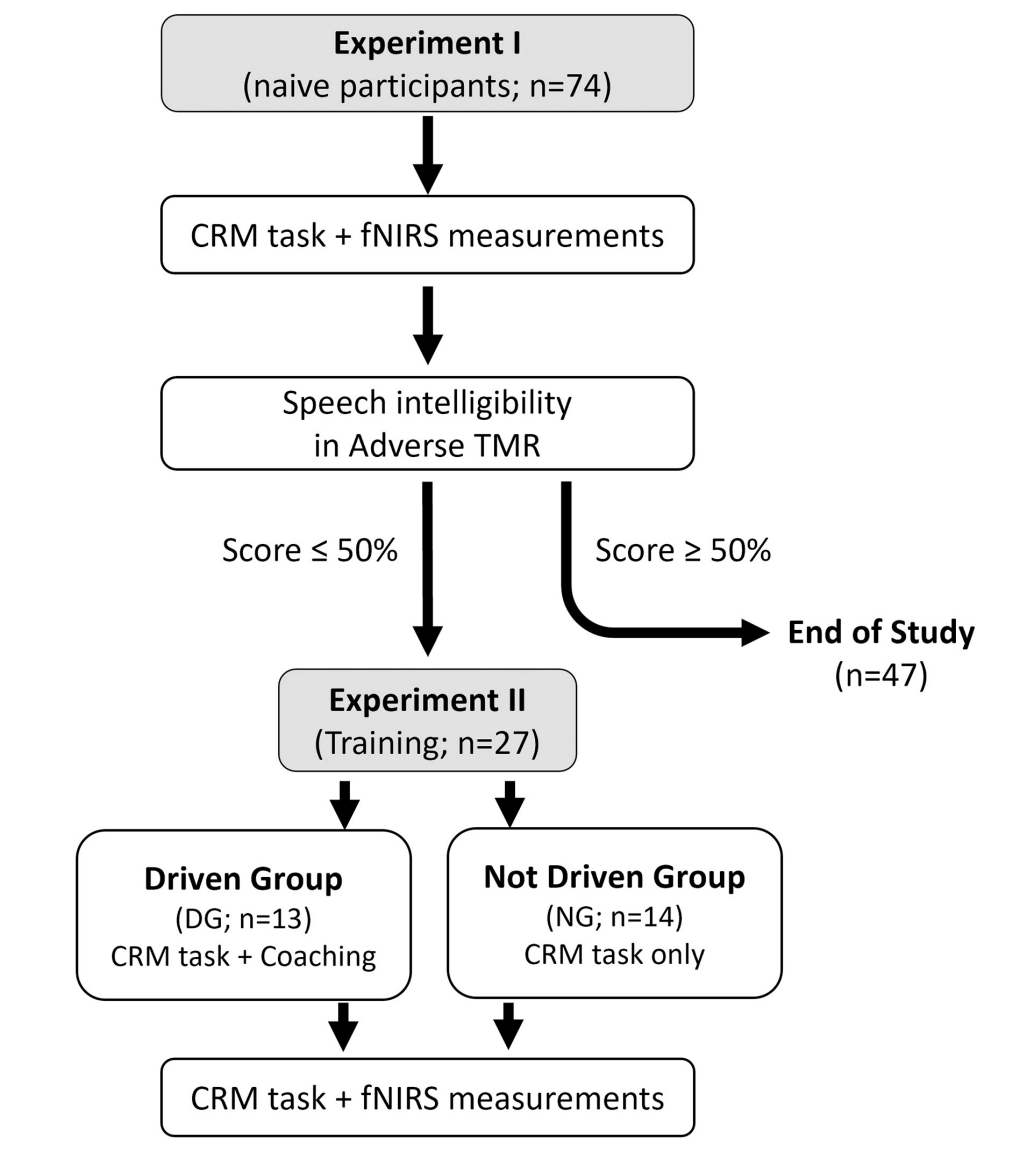
Schematic of the experimental design. The two experiments are illustrated: Experiment I and Experiment II. For each experiment the number of participants and the experimental protocol are specified. The Experiment II also specifies the criterion for selecting the participants, the division into sub-groups and the training strategy used.

To control for task repetition effects, two types of training were tested with two subgroups of participants. The first group, hereafter referred to as the ‘not-driven group’, simply repeated the same task as in Experiment I. The second group hereafter referred to as the ‘driven group’, received explicit verbal information that was meant to facilitate task performance by making it easier for the participants to distinguish the target from the masker (see Fig 6). Specifically, prior to a block of trials, participants from the driven group were informed whether the target voice would be softer (negative TMR condition) or louder (positive TMR condition) than the masker voice. Moreover, auditory feedback, consisting in the target sentence without the masker, was systematically presented after each trial and participants were asked to validate their answer one more time. The presentation of the target sentence in isolation was meant to facilitate learning of relevant perceptual cues and strategies for more easily identifying and hearing-out the target sentence from the target-plus-masker mixture.

#### Training program

All participants were randomly assigned to the not-driven or driven group. The training program was made of three consecutive days of training session (T1, T2 and T3). Each session lasted about 20 minutes and no fNIRS recordings were made during them. At the end of the training program, all 27 participants performed the same task as in the Experiment I with fNIRS recordings. This session, corresponding to the post-training session, lasted 30 minutes.

#### Statistical analyses

*Behavioral analyses*. Mauchly’s sphericity tests on the RAU scores yielded significant results (χ2 = 17.43; *p* < 0.001 for TMR, and χ2 = 8.56; *p* < 0.05 for the TMR-by-session interaction). Accordingly, a two-by-three MANOVA was used to analyze the RAU scores, with Session (Pre- *vs*. Post-training) and TMRs (Adverse *vs*. Intermediate *vs*. Favorable) as within-subject factors and Group (Not-driven Group *vs*. Driven Group) as a between-subject factor. All post-hoc comparisons involved Tukey’s honestly significant difference (HSD) tests. In addition, in order to test whether the trained participants had reached the best performers’ accuracy of Experiment I, we also ran two-sided multiple t-test comparisons between the 27 best performers of Experiment I (Best Pre-test group) and the 27 participants of Experiment II, before training (Pre-training) and after training (Post-training). *P-*values were corrected using the sequentially rejective multiple test procedure [71]. All behavioral statistical analyses were performed using the software STATISTICA^TM^ (V10) from StatSoft® and a significant threshold was set to 0.05.

*fNIRS analyses*. Task activation and relationship between cortical activity and speech intelligibility scores were performed identically to Experiment I. Since behavioral analyses showed no group differences or interaction with it (see Results section), all fNIRS results presented hereafter refer to the whole population (driven + not-driven groups).

*Training effects on the relationship between cortical activity and speech intelligibility scores*. In addition to the fNIRS analyses carried out identically to Experiment I, we have tested a training effect over the relationship between speech intelligibility and brain activations with a robust regression analysis between delta accuracies and delta fNIRS activations (i.e., differences between Post-training and Pre-training sessions). The *robustfit* function from Matlab^®^ with the bisquare weight function and default tuning constant value (i.e., 4.685) was also used.

### Results

#### Behavior

**Fig 7:**
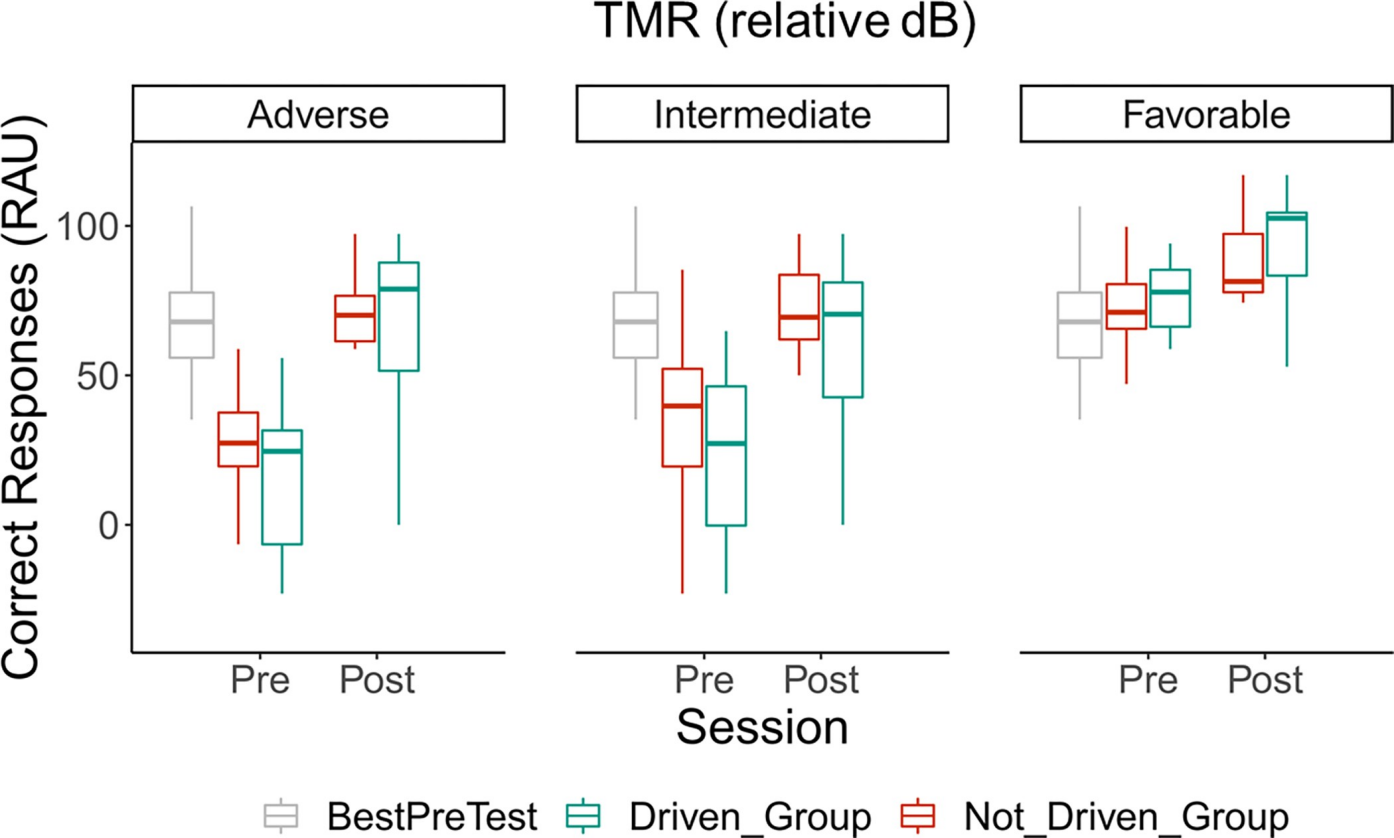
Speech intelligibility scores (RAUs) for each TMR (adverse, intermediate, favorable), training session (pre, post), training group (driven, not driven) and the 24 best performers of Experiment I (BestPreTest). Boxplots show medians (horizontal bars) and interquartile ranges (boxes). Whiskers reach from the lowest to the highest observed value within the 1.5 interquartile range.

The two different training types did not lead to any speech intelligibility difference between groups as attested by the MANOVA (*p* = 0.09 in best case; see Fig 7: driven group and not driven group scores). Similarly, the training type did not interfere with the TMR condition. These results indicate that the two types of training did not lead to any post-training behavioral differences. However, a significant interaction between TMR and the training session was found (F(2,52) = 12.14; *p* < 0.001). As Tukey’s HSD post-hoc tests showed a significant positive training effect for all three TMR conditions (*p* < 0.001 for all TMRs), a graphical interpretation of this interaction suggests a smaller improvement after the training for the favorable condition compared to the two others. Consistently, a main effect of the training session was also found, corresponding to a global significant increase in speech intelligibility after the training (M_pre_ = 41.25, SD = 3.18; M_post_ = 77.20, SD = 3.80; F(1,26) = 72.2; *p* < 0.001).

When compared to the best performers of Experiment I, t-tests revealed that pre-training scores of participants from Experiment II were lower within the adverse (*p* < 0.001; *t* = 9.05) and the intermediate conditions (*p* < 0.05; *t* = 2.73), but not within the favorable one (*p* = 0.13; *t* = 1.55). The same t-tests comparisons for post-training session showed that participants’ scores of Experiment II reached higher accuracy scores in the favorable condition than the best performers of Experiment I (*p* < 0.05; *t* = -3.01).

#### fNIRS

**Fig 8:**
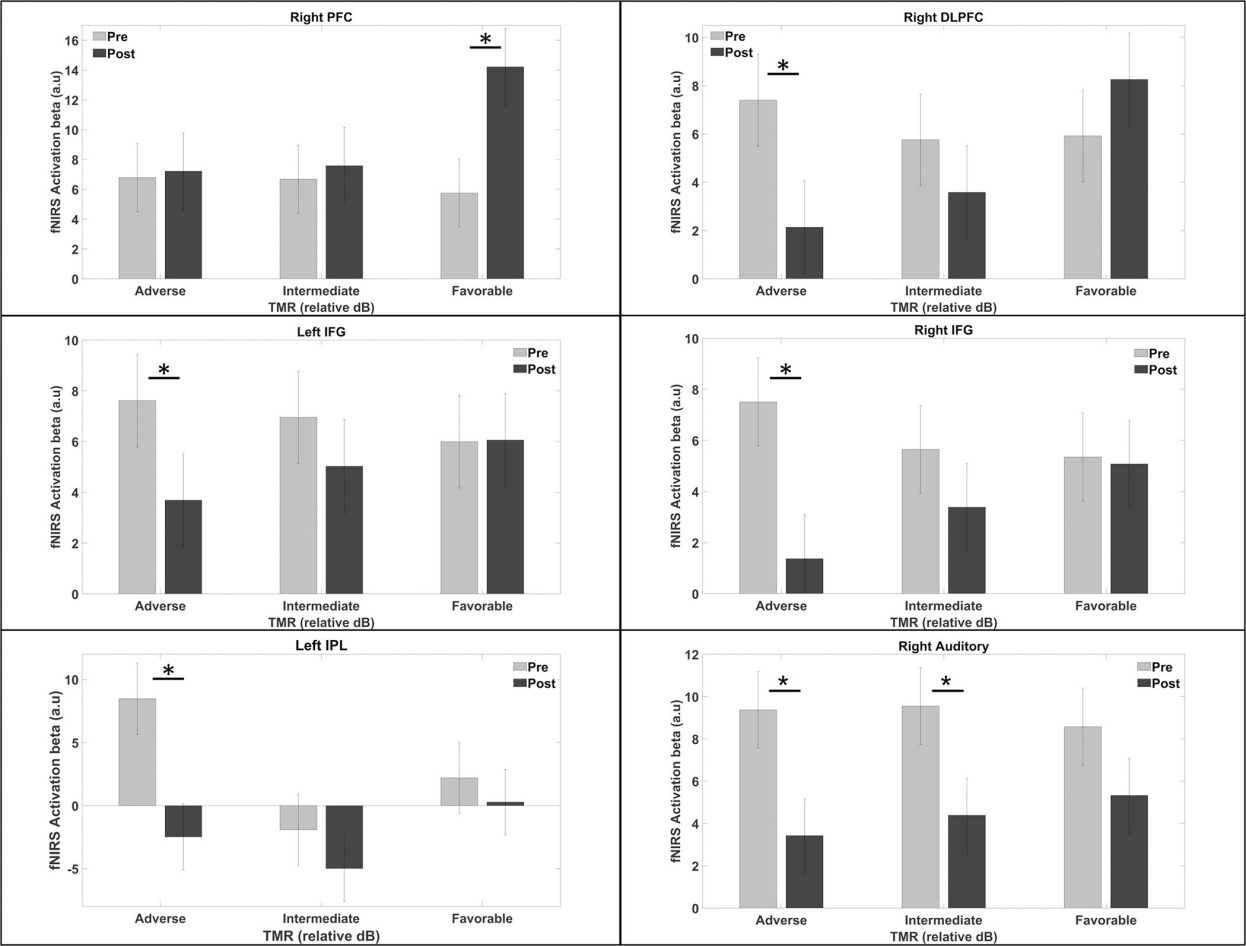
Effects of training on cortical activations for each TMR condition. Pre: Pre-training session; Post: post-training session. * *p* < 0.05 (FDR corrected).

*Task activation*. To investigate the brain correlates of the behavioral improvements following the training, T-tests between pre and post training sessions were carried out. The results showed significant activation decrease in bilateral IFG, right DLPFC, right auditory cortex and left IPL after training (*p* < 0.05; FDR corrected) for the adverse-TMR condition (see Fig 8). These contrasts also revealed a decrease of the right auditory cortex activation following training for the intermediate-TMR condition. This trend of decreasing cortical activity was instead reversed in the right PFC for the intermediate-TMR condition where the cortical activity increased after training.

*Relationship between cortical activation and intelligibility scores*. Contrary to what we found in Experiment I no significant correlation has been found between fNRIS activation and speech intelligibility score in any ROI and condition after the training.

**Fig 9:**
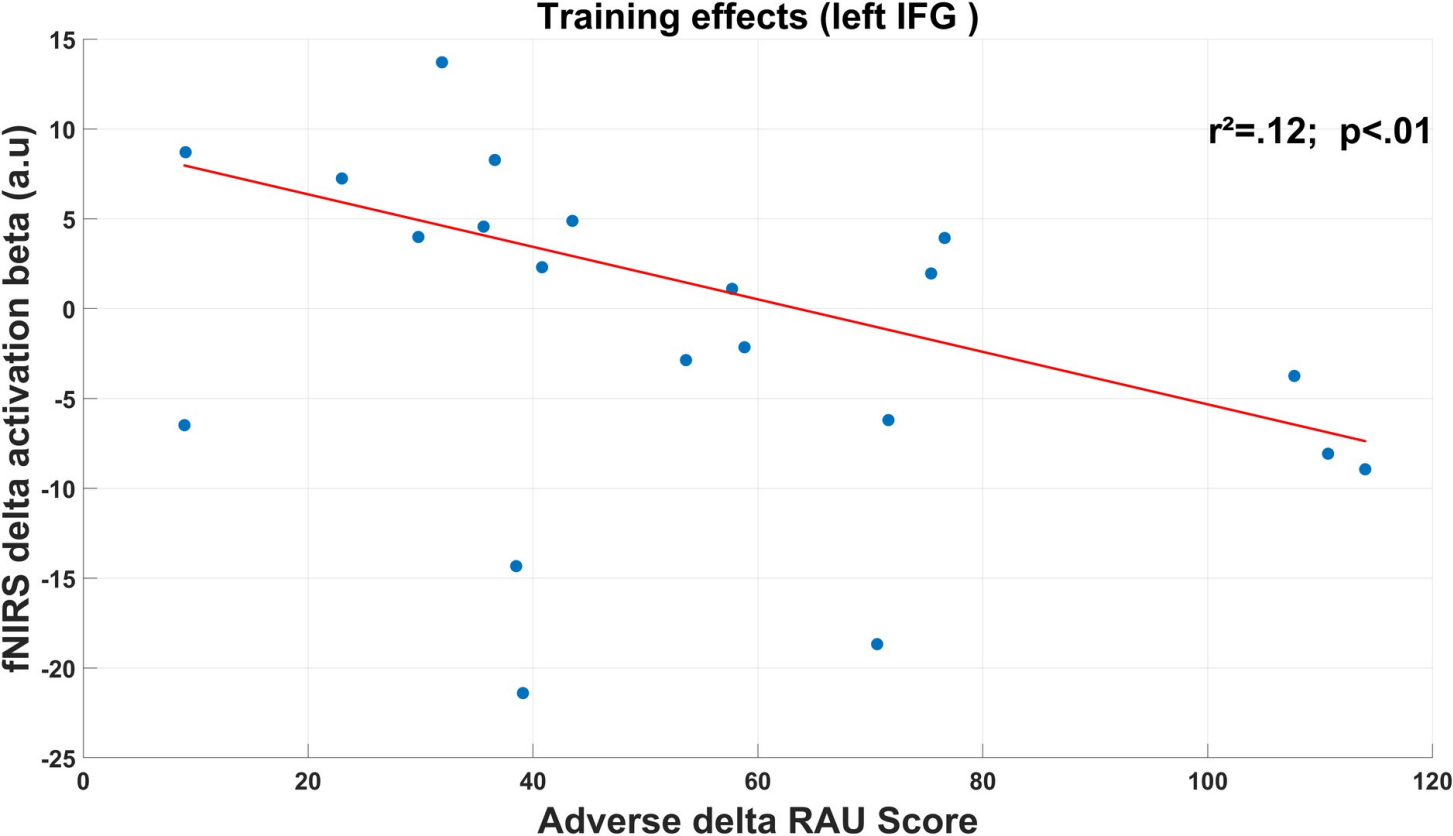
Relationship (robust regression) between training effects over the speech intelligibility (RAU score) in the adverse condition, and training effects over the left IFG activity (fNIRS activation). For both measures, the delta corresponds to the difference between Post-training and Pre-training session values.

Taking into consideration the training effects however, we found a significant negative correlation between behavioral improvements and cortical activity. This effect was only observable for the left IFG and only for the adverse condition, in which we found that the higher the increase of speech intelligibility, the larger the decrease in the left IFG activity (see Fig 9).

### Discussion II

#### Training effects over the speech intelligibility

The findings of similar, statistically significant, improvements in behavioral performance in the not-driven and the driven groups indicate that the provision of explicit instructions and feedback regarding response correctness did not yield greater performance improvements than those afforded by supplementary task practice alone. The improvements observed may have been due, at least in part, to further learning of differentiating the talkers’ voices characteristics by the participants [72]. In addition, or instead, participants may have developed their own strategy to achieve the task [36], probably based on inhibitory control.

In the present study, once the two voices are segregated, the listener has to select the target voice and inhibit the masker voice. Besides, in the adverse condition, the masker voice is louder and therefore easier to select. However, the listener has to inhibit this natural trend in order to focus on the weaker target voice. Thus, inhibitory control seems to be crucial for good speech intelligibility in our informational masking task. The development of an efficient inhibitory control has already been demonstrated during a short period of training without feedback or explicit instructions for well-known inhibition tests (e.g., Go/No-Go test, Stop-Signal task, Stroop task*)*, in EEG [73] and fMRI [74] experiments.

#### Training effects over the cortical activity

We observed that the training led to a decrease of bilateral IFG, right DLPFC and right auditory cortex activities within the adverse-TMR condition, as well as a decrease of right auditory cortex activity in the intermediate-TMR condition. This contrasts with previous studies and our first experiment results that showed greater cortical activation associated with higher speech intelligibility [38, 40]. We can argue however, that measurement of cortical activity during the first training sessions would have probably shown greater activation of IFGs that would have subsequently decrease due to automatization mechanisms. Previous studies have found such initial cortical activity increase, followed by a progressive decrease during training [45, 75]. In Qi et al.’s study [45], the authors measured the cortical activity during a discrimination tasks with speech (Mandarin) tones and sinewave tones, before and after intensive Mandarin course (i.e., listening, reading and writing) in native English-speakers. They showed that participants exhibiting a larger decrease of right IFG activity showed better long-term retention of Mandarin language. A similar pattern–with an increase followed by a decrease of IFG activity during training–has also been observed in an fMRI study using a visuo-spatial working memory task [75]. Finally, decreased IFG activity has also been found in studies involving inhibitory-control tasks [73, 76].

The role of inhibitory control in the task is the second major difference between our study and previous studies that reported better speech intelligibility associated with a higher IFG activation across conditions. Indeed, we used a speech-in-speech task with two talkers producing intelligible sentences, whereas most of the previous studies used degraded speech with only one talker [38, 40]. As a consequence, the inhibition of the loud masker talker, that plays a major role in our task achievement, is not required in the former studies since it doesn’t exist.

At the neural level, inhibitory control has been associated with a consistent network of brain areas involving mainly the frontal cortex and more specifically, the bilateral IFG [63, 74, 77–79]. Inhibitory control belongs to a domain-general system that manages the ability to inhibit interference from task-irrelevant information [65, 80]. Chavan et al. [74] for instance, used a Go/No-Go proficiency test to study the effects of a two-week training on frontal top-down inhibitory control mechanisms. They found that behavioral improvements were associated with decreased activities in bilateral IFG, consistent with our training results. They argued that participants presented a minor engagement of neural populations involved in the inhibitory process to achieve the task.

As an explanation, some authors suggest that neural modifications (e.g., synaptic weight updates), leading to more efficient and lower neural activity, would occur during the training [81]. We suggest now that similar mechanisms were involved in the present study, and that it may explain the decreased activity of the IFG following the training.

#### Training effects over the relationship between speech intelligibility and cortical activity

Finally, one interesting result is the fact the relationship between speech intelligibility scores and cortical activations observed in Experiment I was no more significant in Experiment II. This effect may be explained by 1) the decrease in variability after the training (which was expected) and 2) a lower sample size than Experiment I. Nevertheless, analyzing this relationship in terms of variation between pre- and post-training sessions (i.e., delta performance and delta activation) we found that participants who benefited the most from the training in the adverse-TMR condition were those who reduced the most their left and right IFG as well as left auditory cortex activities. Along with the results from Experiment I, this is additional evidence that some automatization mechanisms in the left IFG may rapidly take place within the speech-in-speech task to provide better speech intelligibility.

## General discussion

The present study aimed to investigate the neural mechanisms underlying the ability of human listeners to selectively attend to, and understand, a talker’s voice in the presence of a competing voice—a particular case of a more general ability, sometimes referred to as ‘cocktail party’ listening. To address this question, two complementary approaches were used. In a first experiment (Experiment I), correlations between behavioral performance and cortical activity were assessed for three conditions, corresponding to different TMRs, yielding different levels of task difficulty. For the adverse-TMR condition only, correlations were observed, indicating greater cortical activity with higher performance. Importantly, for some participants, performance was lower in this adverse-TMR condition than in the two others, more favorable TMR conditions (as expected based on the level of the masker). For other participants, performance was higher in the adverse-TMR condition than in the intermediate-TMR condition. The latter arguably took advantage of the sound level difference between the target and the masker sentences to identify the target. Consequently, a second experiment (Experiment II) used a different approach to further investigate the relationships between task performance and cortical activity. In this approach, training, specific task instructions, and feedback were used to improve the performance of participants who had initially performed poorly in the adverse-TMR condition in Experiment I. Changes in cortical activity were observed, related to changes in behavioral performance with training, providing further evidence for the existence of relationships between these two variables. Together, these two approaches, observational (Experiment I) and training (Experiment II), provide additional support for the hypothesis proposed in earlier studies [14], that left IFG is involved in challenging listening situations.

This leaves the neural activity related to masker inhibition as the most plausible explanation for the combined results of Experiment I and II. By associating learning with synaptic weighting updates and lower neural activity [81–84], the neural efficiency theory provides a comprehensive framework for the dual observation of a better performance and a lower brain activity in terms of a lower cognitive engagement. In line with the assumptions of this theory, Bless et al. [81] found that training in a dichotic task led to better performance and lower cortical activity in brain regions associated with selective auditory processing. A decrease of neural activity resulting from training likely results in cognitive-resource savings that can be used subsequently to perform concurrent tasks. This could be assessed, for instance, in future studies using a dual-task paradigm.

Finally, the multi-talker task used in this study involves inhibition of the masker’s voice to focus on the talker’s voice, suggesting that training benefits may stem from better inhibitory control. Indeed, the inhibitory control is particularly relevant here since the louder masker in the adverse TMR condition is much easier to report than the target talker. Hence, the listener has to struggle against this natural trend to perform correctly. Nevertheless, a broader role of inhibitory control in generic speech-in-speech task has to be confirmed. The crucial role of inhibitory control may be limited to multi-talker situations with large level differences between talkers. For instance, it is expected that no natural trend would be in favor of a given talker if talkers differ only by their gender. However, inhibitory control would help, at least, to limit the interference of the masker talker in any multi talker situations [85]. Future studies could target the role of inhibitory control thanks to specific protocols. For instance, an experiment could reinforce the inhibitory control by training with a non-auditory task, and then test whether that training can improve the speech-in-speech intelligibility. Reciprocally, other experiment could test the effect of speech-in-speech training on non-auditory measurement of inhibitory control.

The present findings argue that the improvement in speech-in-speech intelligibility can be mediated by a cognitive ability, namely the inhibitory control. Since that ability is not auditory specific, it could be trained by non-auditory tasks (e.g., serious games) and then benefits patients with clinical or subclinical auditory lesions. Moreover, as suggested by the decrease of the left IFG activity following the training, such training should spare cognitive resources and therefore be more beneficial for the elderly or the workers submitted to cognitive constraints.
